# A nose for trouble: ecotoxicological implications for climate change and disease in Saiga antelope (*S. t. tatarica*)

**DOI:** 10.1007/s10653-024-01874-y

**Published:** 2024-02-17

**Authors:** S. T. Mullineaux, J. M. McKinley, N. J. Marks, R. Doherty, D. M. Scantlebury

**Affiliations:** 1 School of Natural and Built Environment, Queen’s University Belfast, Belfast, Northern Ireland UK; 2 School of Biological Sciences, Queen’s University Belfast, Belfast, Northern Ireland UK

**Keywords:** Saiga mass die-off, Smog, Compositional data analysis (CoDA), Epidemiological, Environmental pollution

## Abstract

In recent decades, Saiga antelope (*Saiga t. tatarica*) mass die-offs have become more common. The mass die-off of 2015 in central Kazakhstan, recorded 140,000 individual deaths across multiple herds. Previously, research has shown atmospheric humidity, the bacterium *Pasteurella multocida* serotype B, and resultant haemorrhagic septicaemia, were the primary cause. However, other synergistic factors may have impacted this process. Here we use a multivariate compositional data analysis (CoDA) approach to assess what other factors may have been involved. We show a pollutant linkage mechanism where relative humidity and dewpoint temperature combine with environmental pollutants, potentially toxic elements (e.g., Hg, As), complex carbon compounds (e.g., Acetone, Toluene), and inorganic compounds (e.g., CHx, SO_2_) which affected the Saiga during the calving season (start and peak) and at the onset of the mass die-off. We suggest a mechanism for this process. Upon arrival at their carving grounds, the Saiga experienced a sudden precipitation event, a spike in temperatures, and resultant high humidity occurs. The infectious bacterium *P*. *multocida* serotype B then spreads. Further, environmental pollutants contained within steppe soils are released to the air, forming localised smog events, these synergistically combine, and mass die-off occurs.

**Supplementary Information:**

The online version contains supplementary material available at 10.1007/s10653-024-01874-y.

## Introduction

### Saiga antelope physiology and mass die-off phenomena

Mass die-off phenomena or mass mortality events , occur when a significant percentage of a population is lost within a short period of time. These occurrences are preceded by an environmental anomaly, which then synergises with environmental health factors, and cascades to an event (Fey et al., 2015). Such events are uncommon but have become more frequent as climate change exacerbates (Fey et al., 2015; Robinson et al., 2019). Mass die-off events are not the same as mass extinction events (significant percentage loss of biodiversity) or ecosystem collapse events (collapse of trophic structures). Mass die-off/ mass mortality events are species or genera-specific, with collapses noted in a population at a specific point in time and space. Evidence of these occurrences can be seen in both the present and the past.

Saiga antelope physiology is highly specialised. This species evolved to cope with a range of climatic extremes, arising from Ice-age ancestors (Campos et al*.*, 2010; Jürgensen et al., 2017) and remain highly migratory to the modern-day (Nadachowski et al., 2016). Within Kazakhstan the herds migrate from north–south following seasonal fluctuations in grass nutrition that peaks in spring; the species calving season (Singh et al., 2010). The species is threatened by poaching which has caused them to avoid humans (Frey et al., 2007). The nose of Saiga is highly specialised, adapted to remove dust from the air they breathe, and for communication. Males vocally display during the rut Clifford & Witmer, 2004; Frey et al., 2007), and females contact call their calves (Sibiryakova et al., 2017; Volodin et al., 2014).

In 2015, a mass die-off event of Saiga antelope occurred in central Kazakhstan (Betpak-Dala) after a 10-day period of unusually humid and hot weather. Infection of the bacterium *P*. *multocida* serotype B spread, causing haemorrhagic septicaemia, killing at least 140,000 individuals, half the global population of this critically endangered species (Fereidouni et al., 2019; Kock et al., 2018). Post-mortem studies noted the lungs of the Saiga were congested, suffering from haemorrhages and mild emphysema. Further congestion and ill effects were documented in the heart, liver, kidney, and gallbladder. The pathogen *Clostridium perfringens* was noted as a possible secondary cause (Wolfs, 2021).

### Environmental and industrial factors within Kazakhstan

Kazakhstan contains large areas of agricultural land and has seen large-scale industrilaisation in energy, petrochemical, and metallurgical industries. There has been a measurable increase of potentially toxic elements in urban soils (Iztileu et al., 2013) and air pollutants have also increased. In the city of Almaty (southern Kazakhstan) particulate and NO_2_ pollutants from coal combustion were shown to be above WHO annual limits, and SO_2_ and CO were also present (Kerimray et al., 2020).

The grassland steppe soils, and climate of this region are unique. Comprised of steppe, steppe-forest, steppe-desert, and desert latitudinal zones with a highly seasonal continental climate (Pachikin et al., 2014). There has been widespread soil degradation from intensive agriculture, and levels of soil pollutants have increased throughout Kazakhstan (Almaganbetov & Grigoruk, 2008). In Betpak-Dala, where the 2015 mass die-off occurred, soils are classified as either brown, or gray-brown desert soils (arenosol soils). These soils have high levels of carbonates, low organic content, superficial porous crusts, and can form complexes with solonetz (Sodium rich) soils that contain soluble salts in the lower parts of their profiles (Pachikin et al., 2014; Yapiyev et al., 2018). Aeolian deposition of pollutants onto these geochemically complex soils with little hydrological activity is likely to cause the storage of these chemical agents.

### Proposed pollutant linkage mechanism for environmental synergy and compositional approaches

**Fig. 1:**
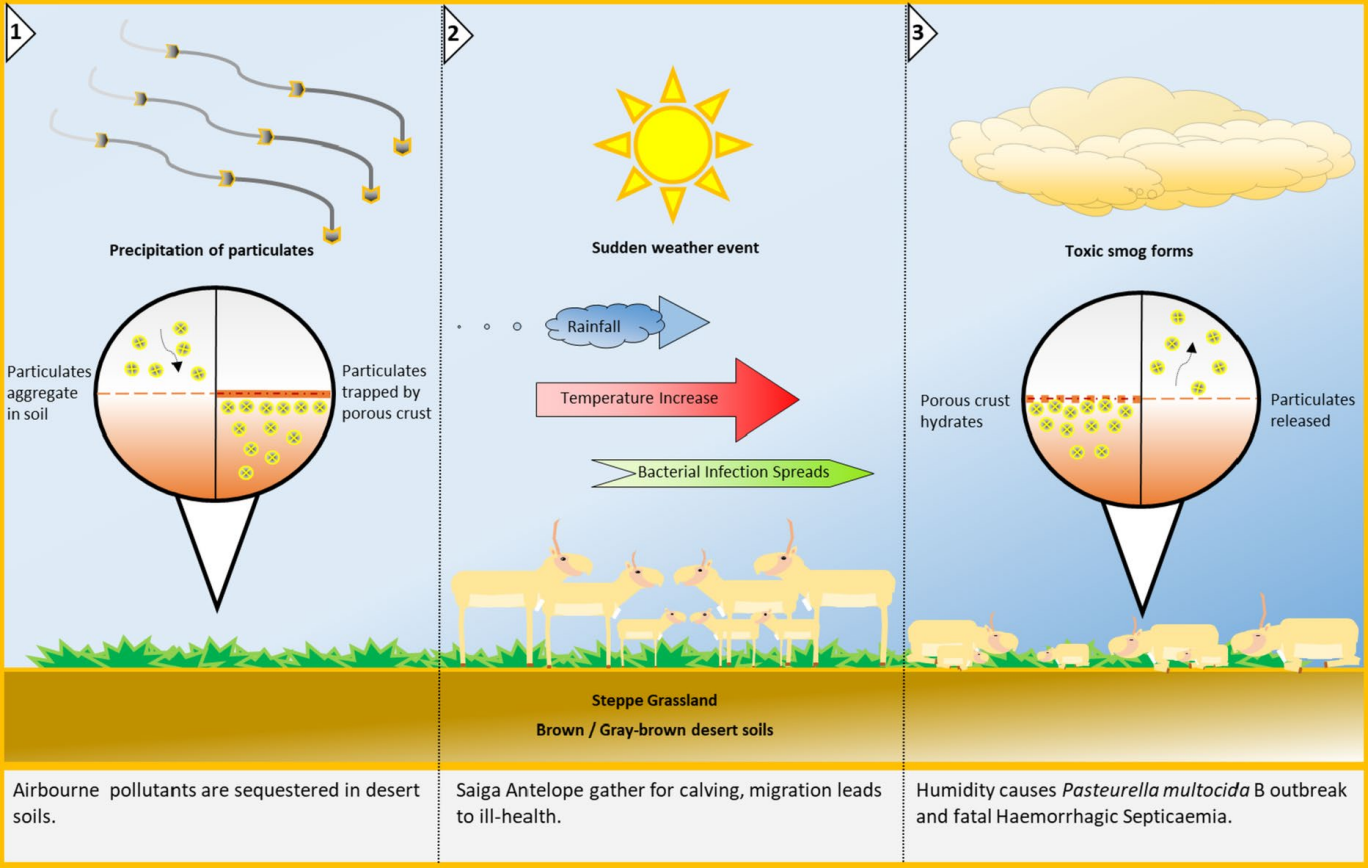
Proposed mechanism for the retention and release of pollutants in the steppe

We suggest a novel environmental mechanism for the mass die-off phenomena events that occurred in Saiga (Fig. 1). This model has anthropogenic sources, mainly environmental pollutants from industrial and urban activity. Abiotic pathways allow the environmental pollutants to become active, like temperature and humidity. Then the biotic factors affecting the receptor (Saiga) such as population stress, density, and infectious disease. The factors combine into a pollutant linkage model of ‘Source-Pathway-Receptor’ that is quantified using a compositional approach (Waldschläger et al., 2020).

**Fig. 2:**
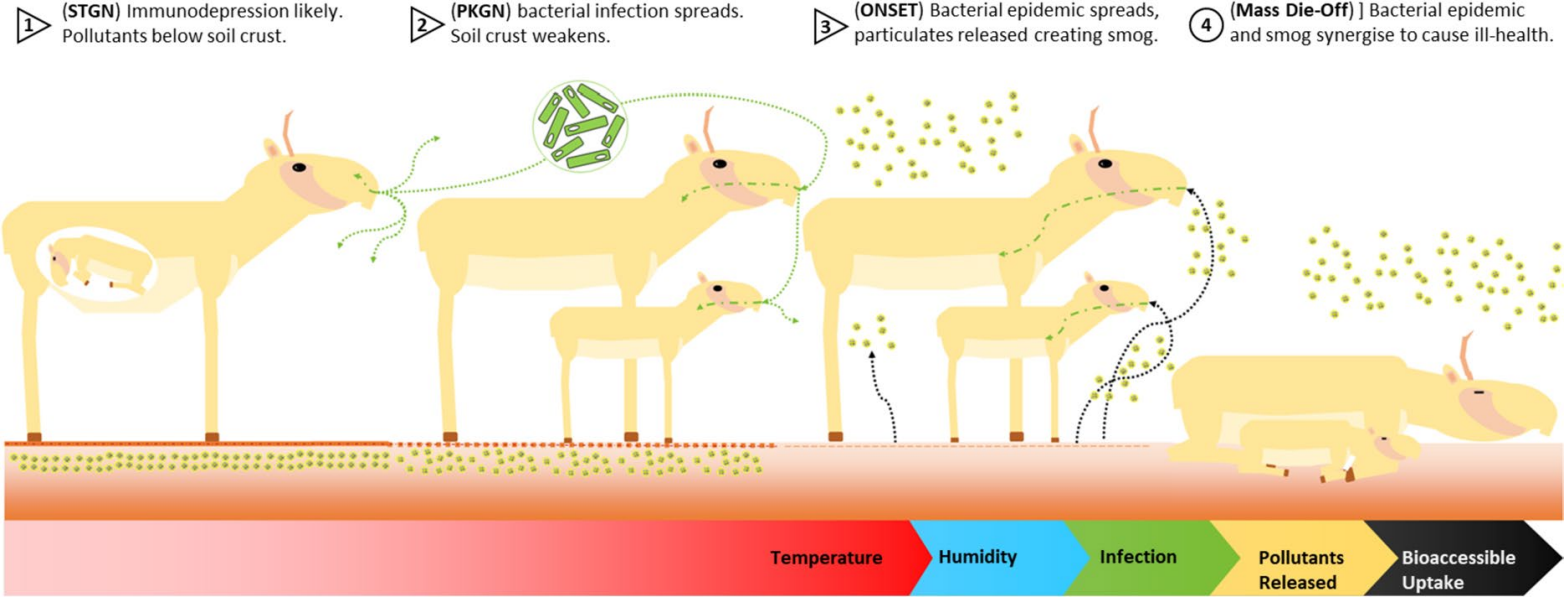
Proposed mechanism for the effects of migration, pregnancy, pollutants, smog, and bacterial infection, given the Saiga’s specialised anatomy. STGN: start of calving season; PKGN: peak of calving season; ONSET: onset of mass die-off event

We use a compositional data analysis (CoDA) approach to consider the proportional relationships between multiple environmental pollutants, and conceptualise their synergistic activities (Mullineaux et al., 2021a, 2021b). This treats variables as co-related entities, under a zero-sum constraint. Which allows the environmental pollutants to be considered as parts contributing to a whole. Both the centred log ratio (clr) and the isometric log ratio (ilr) transformations are used. The compositional relationships between pollutants and meteorological factors are elucidated and the potential impact on Saiga antelope in Betpak-Dala is assessed (Fig. 2). The central question is what role, if any, could environmental pollutants have played in the Saiga mass die-off events of 2015, and can the compositional approach elucidate a possible pathway?

## Materials and methods

### Data for the analysis

#### Saiga antelope mass die-off and meteorological data

All data for this study were downloaded from published sources. Mass die-off events were recorded as a binary variable (0 and 1, n = 94) nested in both site (space) and year (time) in the Baptuk-Dala region. This was downloaded from the NERC data repository (https://doi.org/10.5285/912ea336-ac90-418f-be6a-7ae226e167e9) (Robinson et al., 2018). The meteorological data was also included in this dataset (Kock et al., 2018; Robinson et al., 2018). This data has three parameter states: start of calving season, peak of calving season, and onset of the mass die-off event. The original analysis included a range of time-windows across different spans of days. For this study all three parameter states were assessed but only the 10-day average was used, as shorter time-periods did not capture the variation needed to test the proposed mechanism. The meteorological data contains variables for temperature, rainfall, soil–water, wind-gust, humidity, and dewpoint; with both humidity and dewpoint being related to potential smog formation.

#### Ecotoxicological data and the compositional approach

The environmental pollutant data was collected from the department for the environment of Kazakhstan. The data represents the time-period of 2000–2015 and is expressed as tons per year (Ministry of National Economy of the Republic of Kazakhstan Committee on Statistics, 2014). The pollutants included are the potentially toxic elements, complex carbon compounds, and gaseous industrial pollutants.

The compositional approach was chosen to account for the proportional relationships of the air pollutants in particulate form. Also, the synergistic nature of their interactions, and the compositional signatures they would create by aeolian deposition processes upon soils. Both the Centered Log-Ratio (clr) approach, and the Isometric Log-Ratio (ilr) approach were applied to the pollutant data in this study. The clr transformation accounts for the compositional (proportional) relationships between variables whilst retaining the geometry of the original data table. Whilst the ilr transformation considers the orthogonal relationship between a pair of variables as part of a larger cluster or sub-composition, so the ratio between two environmental pollutants can be visualised as a single variable (Egozcue et al., 2003; Filzmoser & Hron, 2009).The transformed data were compared to the meteorological data to identify if the mass die-off mechanism proposed in Fig. 2 could be verified by the statistical analysis.

### Statistical analysis

#### Logistic regression

**Fig. 3:**
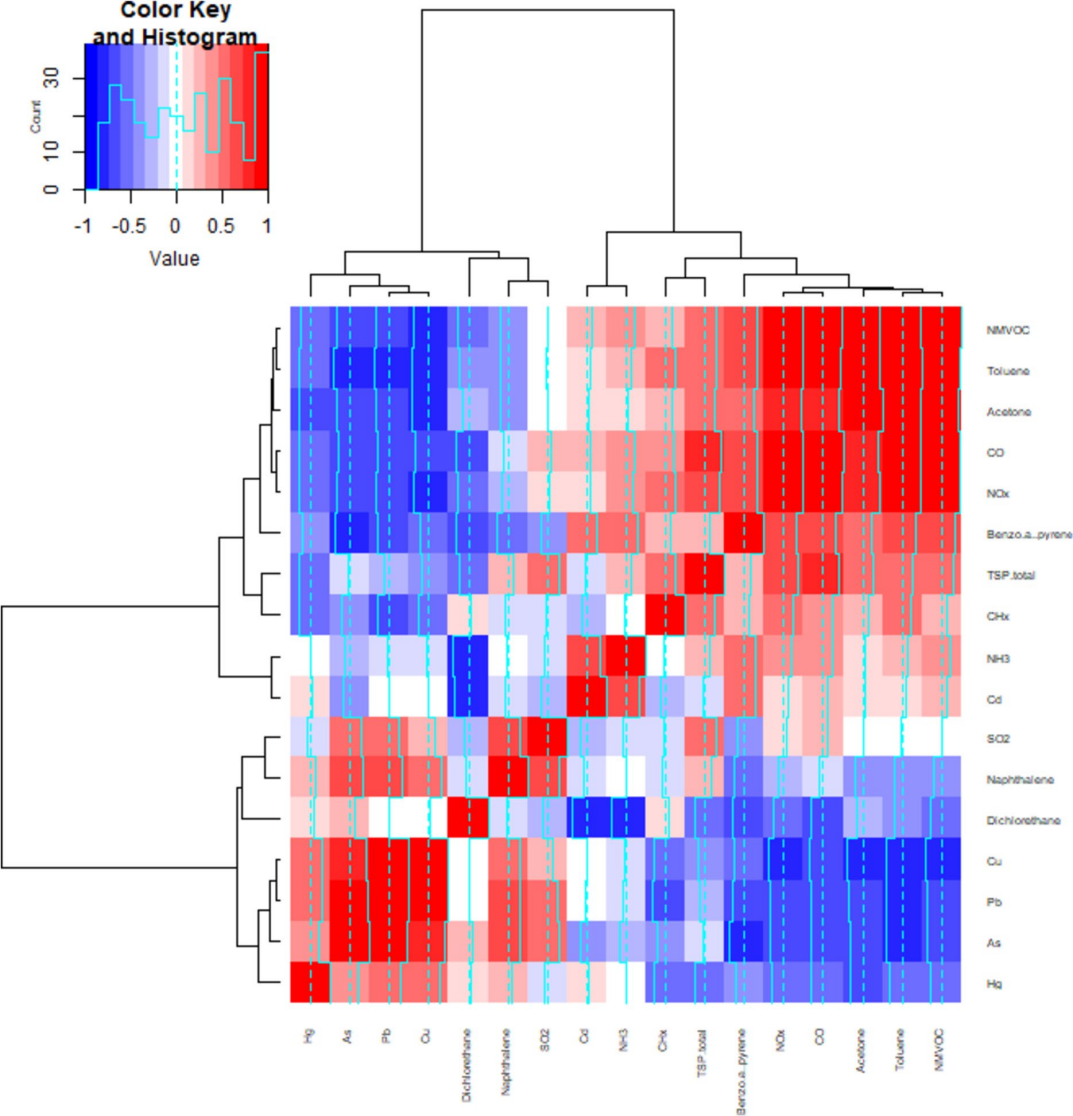
Complex heatmap-dendrogram of environmental pollutants, tonnes per year between 2000 and 2015 (clr transformed), pairings used for ilr balances (colour key and histogram indicates spearman’s correlation value)

Logistic regression models of mass die-off events (from 2000 to 2015) were modelled against compositionally transformed pollutant data and the meteorological variables. The meteorological variables from the start of the calving season (STGN), the peak of the calving season (PKGN), and the onset (ONSET) of each die-off event. The clr and ilr compositional transformations were used on the air pollutant data and modelled to infer their interactions with Saiga antelope mass die-off events (Egozcue et al., 2003; Filzmoser & Hron, 2009; Hernandez et al., 2017). Figure 3 shows how the clr transformed environmental pollutants relate to each other compositionally in a complex heatmap (Mullineaux et al., 2021a). These clusters were used to inform the ilr sub-compositions. In turn, cluster pairings in the complex heatmap are the ilr balances used and are denoted by: “A_B”, where “A” and “B” are pollutants, and the “underscore: _” represents the balance shorthand. Mathematically, a balance corresponds to the difference in means of the log-transformed abundances between two sub-compositions (McKinley et al., 2020). The use of balances allows components whose relative abundance is associated with an occurrence of an eco-environmental event to be identified.

#### Principal component analysis

In the final phase of the analysis, principal component analysis (PCA) was used to isolate which combinations of compositionally transformed pollutant variables, and meteorological variables from each time-point were synergistic. Further logistic regressions were conducted on the principal components 1–3, of each scenario (STGN, PKGN, and ONSET, paired with either clr or ilr transformed data) and the mass die-off event data. This approach was used to verify the synergistic effects of environmental pollutants and meteorological factors, as proposed above.

### Variables used in analysis

The sources (pollutants) included in this analysis are, the potentially toxic elements): Lead (Pb), Cadmium (Cd), Mercury (Hg), Copper (Cu), and Arsenic (As) (Cu does have essential functions at low levels) (World Health Organization, 1996). The complex carbon compounds: Toluene (C7H8), Benzo-α-Pyrene (C20H12), Naphthalene (C10H8), Dichloroethane (C2H4Cl2), and Acetone (C3H6O). The industrial gaseous pollutants: sulphur dioxide (SO_2_), Nitrous oxides (NOx), Non-methane volatile compounds (NMVOC), Ammonia (NH_3_), Carbon Monoxide (CO), uncategorised Hydrocarbons (CHx), and total suspended particulate (TSP) (TSP represents potentially undocumented pollutants and documented pollutant complexes). The pathways or meteorological variables from (Robinson, 2018) are nested in both site (space) and year (time). Therefore, the interpretation of these models is directly nested in the occurrence of a mass die-off event. In the variables below, capitalisations refer to the terms as follows: TOT: total, PRECEP: precipitation days, GCPP: GC precipitation days, SOILWATER: soil water, MAX: maximum, MIN: minimum, TEMP: temperature, AV: average, DIFF: difference, DEWP: dewpoint, AVMN: average-minimum, MXMN: maximum of minimum, AVMX: average-maximum, MXMX: maximum of maximum, RHUMID: relative humidity, and WINDGUST: wind-gust.

## Results and discussion

The results from the initial phase of analysis comprise the Saiga Antelope die-off events, which took place at different site locations, between the years of 2000–2015. This is the dependent variable. The environmental pollutants released as tonnes per year between 2000 and 2015, representing the aeolian deposition signal onto soils, and the meteorological variables (10 days) from each phase: STGN: start of calving season, PKGN: peak of calving season, and ONSET: onset of mass die-off are the independent variables. Given the number of variables in this analysis, thresholds of importance for independent variables are presented in three categories. High degree of significance (significant p-value and Akaike Information Criterion (AIC) value of less than 50), moderate degree of significance (significant p-vale and AIC value less than 60), and low degree of significance (significant p-value and AIC value greater than 60).

### Logistic regression clr and ilr output

The logistic models for both the clr and ilr transformed pollutant variables highlight significant relationships between mass die-off events and environmental pollution signals. The clr transformed pollutant data (Table 1), shows a high degree of significance for NMVOC and CHx, a moderate degree of significance for Acetone, and a low degree of significance for Cd, Hg, Naphthalene, Dichloroethane, NOx, NH_3_, CO, and TSP. Toluene also shows a low AIC value, but the model is not significant. The ilr transformed pollutant data shows a high degree of significance for As_Pb and, Pb_Cu, a moderate degree of significance for CHx_TSP, and Benzo-α-pyrene_NOx, and a low degree of significance for Dichloroethane_Naphthalene, Naphthalene SO_2_, and Cd_NH_3_ (Table 1).The observations for mass die-off events are nested primarily in site but also in time and a 1 score denotes the occurrence of a mass die-off event observed at a site in 2015. The atmospheric environmental pollutant data is from national statistics data, nested in time (year) representing an aeolian depositional signal in this analysis. Thus, potential error is introduced into these models through pseudo-replication of the environmental pollutant data (the independent variable). The analysis was thus conducted to allow for comparisons to the meteorological data, which varies across both site (space) and year (time) allowing co-variance across the models to occur. Both data types are incorporated into the PCA analyses for further comparison and verification of the noted below.

**Table 1:** Logistic regression, p and AIC values (NA shows models which did not converge) for clr and ilr transformed data, decision heatmap-dendrogram for ilr pairs can be seen in Fig. 3

Pollutant	*P* Value	AIC
Pb_clr	0.18401	80.89063
Cd_clr	0.00779	60.33722
Hg_clr	0.03689	78.00889
Cu_clr	0.21694	81.10002
As_clr	0.99840	NA
Toluene_clr	0.06748	38.76899
Benzo_α _pyrene_clr	0.99547	NA
Naphthalene_clr	0.04885	76.10781
Dichlorethane_clr	0.00016	66.52192
Acetone_clr	0.00016	56.57529
SO_2__clr	0.97488	83.12056
NOx_clr	0.00053	66.12081
NMVOC_clr	0.00031	45.69836
NH_3__clr	0.00976	73.02341
CO_clr	0.00057	67.30915
CHx_clr	0.00626	41.54626
TSP_total_clr	0.02781	77.37662
Hg_As_ilr	0.99869	NA
As_Pb_ilr	0.02660	25.41473
Pb_Cu_ilr	0.00546	21.13527
Dichlorethane_Naphthalene_ilr	0.00026	67.90870
Naphthalene_SO_2__ilr	0.00018	66.81173
Cd_NH_3__ilr	0.02615	63.03972
CHx_TSP_ilr	0.00035	53.49624
Benzo_α _pyrene_NOx_ilr	0.00111	53.78051
NOx_CO_ilr	0.99763	NA
CO_Acetone_ilr	0.18088	81.00872
Acetone_Toluene_ilr	0.00817	72.98484
Toluene_NMVOC_ilr	0.99501	NA

*P* value < 0.05 indicates significance, AIC < 50 highly significant, AIC > 50 moderately significant, low degree of significance (significant p-value and AIC value greater than 60), *P* value > 0.05 indicates insignificance

### Logistic regression meteorological output

In the variables below capitalisations refer to the terms as follows: TOT: total, PRECEP: precipitation days, GCPP: GC precipitation days, SOILWATER: soil water, MAX: maximum, MIN: minimum, TEMP: temperature, AV: average, DIFF: difference, DEWP: dewpoint, AVMN: average-minimum, MXMN: maximum of minimum, AVMX: average-maximum, MXMX: maximum of maximum, RHUMID: relative humidity, WINDGUST: wind-gust and 10D denoting the 10-day average. For example, the first variable mentioned below: DEWPAVMN, is dewpoint-average-minimum, the last RHUMID_MAXOFMN10D is relative-humidity maximum-of minimum-10-days.

### Start of calving season

The results for STGN (start of the calving season) show a high degree of significance for DEWPAVMN, DEWPMXMN_10D, DEWPAVMX_10D, DEWPMXMX_10D, AVRHUMID_MAX10D, MAXRHUMID_MAX10D, and MINRHUMID_MIN10D. A moderate degree of significance for GCPP_PRECIP_10D, GCPP_PRECIPDAYS_10D, RHUMID_MEAN10D, AVRHUMID_MEAN10D, MAXRHUMID_MEAN10D, WINDGUST_MNOFMAX10D. A low degree of significance for TOTPRECIP_10D, SOILWATER1_MAXMN10D, MAXTEMP_10D, TEMPAV_10D, AVOFMINTEMP_10D, AVOFMAXTEMP_10D, AVTEMPDIFF_DAY_10D, MXTEMPDIFF_DAY_10D, RHUMID_MAXOFMN10D, and RHUMID_MAXOFMN10D (Table 2). Overall, variables associated with humidity show the highest levels of significance.

**Table 2:** Logistic regression, p and AIC values for meteorological variables at the predicted start of the calving season (STGN), peak of the calving season (PKGN), and onset of the calving season (ONSET)

	STGN		PKGN	
Meteorological Variable	*P* value	AIC	*P* value	AIC
TOTPRECIP_10D	0.04415	79.01023	0.00010	61.64767
GCPP_PRECIP_10D	0.00002	55.78049	0.00008	59.93321
PRECIPDAYS_10D	0.06134	79.38891	0.00004	51.69319
GCPP_PRECIPDAYS_10D	0.00002	53.61655	0.00048	30.04943
SOILWATER1_MAXMN10D	0.02904	77.71028	0.00163	70.43362
MINTEMP_10D	0.68888	82.96035	0.28729	81.98746
MAXTEMP_10D	0.01908	76.77690	0.49884	82.66017
TEMPAV_10D	0.00105	66.43260	0.35189	82.25393
AVOFMINTEMP_10D	0.00198	68.94714	0.00718	74.04754
AVOFMAXTEMP_10D	0.00068	64.47148	0.91186	83.10926
AVTEMPDIFF_DAY_10D	0.01116	75.55278	0.00022	38.26262
MXTEMPDIFF_DAY_10D	0.00682	74.62334	0.02120	77.63435
TEMPDIFF_MINS_10D	0.24159	81.74920	0.85242	83.08663
DEWPAVMN_10D	0.00019	26.56051	0.00101	26.05975
DEWPMXMN_10D	0.00001	40.08802	0.00005	49.63541
DEWPAVMX_10D	0.00004	33.16152	0.00050	28.55735
DEWPMXMX_10D	0.00008	49.92486	0.00010	49.85187
RHUMID_MEAN10D	0.00098	58.76193	0.00303	33.09771
RHUMID_MAXOFMN10D	0.00175	67.98979	0.00014	58.39726
AVRHUMID_MAX10D	0.00043	43.63198	0.00026	49.53737
AVRHUMID_MEAN10D	0.00102	56.13186	0.00230	30.54658
MAXRHUMID_MEAN10D	0.00063	54.49800	0.00174	26.50865
MAXRHUMID_MAX10D	0.00320	28.73120	0.03391	73.83179
MINRHUMID_MEAN10D	0.00472	72.44795	0.00095	40.73497
MINRHUMID_MIN10D	0.00009	43.00484	0.00015	41.87824
WINDGUST_MNOFMAX10D	0.00069	53.28811	0.00659	74.55365
WINDGUST_MXOFMAX10D	0.26012	81.78687	0.33885	82.18335
DIFFTEMP_10D	0.11398	80.63798	0.61336	82.86280

*P* value < 0.05 indicates significance, AIC < 50 highly significant, AIC > 50 moderately significant, low degree of significance (significant *P* value and AIC value greater than 60), *P* value > 0.05 indicates insignificance

### Peak of calving season

The results for PKGN (peak of the calving season) show a high degree of significance for, AVTEMPDIFF_DAY_10D, DEWPAVMN_10D, DEWPMXMN_10D, DEWPAVMX_10D, DEWPMXMX_10D, RHUMID_MEAN10D, AVRHUMID_MEAN10D, MAXRHUMID_MEAN10D, MINRHUMID_MEAN10D, MINRHUMID_MIN10D. A moderate degree of significance for, GCPP_PRECIP_10D, PKGN, PRECIPDAYS_10D, RHUMID_MAXOFMN10D. A low degree of significance for TOTPRECIP_10D, AVOFMINTEMP_10D, MXTEMPDIFF_DAY_10D, MAXRHUMID_MAX10D, and MAXRHUMID_MAX10D (Table 2). Overall, like the STGN analysis, variables associated with humidity show the highest levels of significance.

### Onset of mass die-off

The results for ONSET (onset of mass die-off) show a high degree of significance for, MAXRHUMID_MEAN10D, DEWPAVMX_10D, DEWPMXMN_10D, DEWPMXMX_10D, and DEWPAVMN_10D. A moderate degree of significance for, AVRHUMID_MEAN10D, and GCPP_PRECIP_10D. A low degree of significance for, SOILWATER1_MAXMN10D, WINDGUST_MNOFMAX10D, TOTPRECIP_10D, PRECIPDAYS_10D, MAXTEMP_10D, AVOFMAXTEMP_10D, TEMPAV_10D, and AVOFMINTEMP_10D (Table 3). Overall, like the STGN and PKGN analysis, variables associated with humidity show the highest levels of significance.

**Table 3:** Logistic regression, *P* and AIC values for meteorological variables at the predicted onset of the mass die-off

Onset: of die off event	*P* value	AIC
MAXRHUMID_MEAN10D	0.00042	49.85829
AVRHUMID_MEAN10D	0.00085	54.82576
DEWPAVMX_10D	0.00009	27.27887
DEWPMXMN_10D	0.00002	35.88428
DEWPMXMX_10D	0.00020	42.37481
DEWPAVMN_10D	0.00032	22.73347
SOILWATER1_MAXMN10D	0.01285	75.88268
DIFFTEMP_10DAY	0.57862	82.81723
WINDGUST_MNOFMAX10D	0.00065	65.71371
WINDGUST_MXOFMAX10D	0.30061	82.00164
GCPP_PRECIP_10D	0.00019	58.39094
TOTPRECIP_10D	0.00325	73.17642
PRECIPDAYS_10D	0.00331	73.07322
MAXTEMP_10D	0.02185	77.35463
AVOFMAXTEMP_10D	0.00549	73.39686
TEMPAV_10D	0.00232	70.04311
MINTEMP_10D	0.07725	79.82018
AVOFMINTEMP_10D	0.00095	64.94130
MXTEMPDIFF_DAY_10D	0.49689	82.64282
SNOWMELT_DAYONSET	0.12496	80.66898
TEMPDIFF_MINS_10D	0.66985	82.94048
NDVI_MEAN15D	0.08748	79.87465

*P* value < 0.05 indicates significance, AIC < 50 highly significant, AIC > 50 moderately significant, low degree of significance (significant p-value and AIC value greater than 60), *P* value > 0.05 indicates insignificance

### Congruence between approaches

The results of this analysis are congruent with the prior conclusion. Rainfall and high temperatures caused humidity allowing the bacterium *P*. *multocida* serotype B to spread. Infection in the Saiga herds followed and mass die-off events across multiple sites occurred (Fereidouni et al., 2019; Kock et al., 2018)^.^ Furthermore, from the PCA outputs compositional biplots with families of meteorological variables added shows several environmental pollutants are associated with the meteorological variables; especially those linked to humidity and temperature. Careful consideration is required, given the nature of the data in this analysis.

### Congruence across analyses

The clr and ilr transformations pose different questions and highlight the limitations of this analysis. The clr transformation considers the compositions (environmental pollutants) to have equal degrees of association. Whilst the ilr transformation allows shared associations to be considered but requires prerequisite justification of these relationships (balances). These must be selected a priori. Thus, this was done using the clr complex heatmap in Fig. 3. where patterns between the pollutants can be seen, and clusters inform the balances. Consequently, direct comparisons between the clr and ilr transformations cannot be considered. Though the implications of these comparisons can be discussed with this knowledge in mind (Egozcue et al., 2003; Filzmoser & Hron, 2009).

The clr transformation shows Cd, Hg, Naphthalene, Dichloroethane, Acetone, NOx, NMVOC (Non-methane volatile organic compounds), NH_3_ (Ammonia), CO (Carbon Monoxide), CHx (uncategorised Hydrocarbons), TSP_total (Total Suspended Particulate total), all have a significant relationship with die-off events. NMVOC and CHx show the most significant relationship. In the ilr transformed data As_Pb, Pb_Cu, Dichloroethane_Naphthalene, Naphthalene_SO_2_, Cd_NH_3_, CHx_TtTSP, and Benzo_α _pyrene_NOx show significant relationships with mass die-off events. As_Pb and Pb_Cu show the most significant relationship (Table 1). These results infer a contradiction, as under clr complex carbon compounds and particulates are highlighted. Whilst under ilr, potentially toxic elements are highlighted. In both, complex carbon compounds, NH_3_, NOx, and CO are highlighted, but potentially toxic elements are only highly significant under the ilr transformation. This could be miscellaneous, but this is likely due to a wider environmental or industrial signal (Kerimray et al., 2020).

The meteorological data at each time point considered (STGN, PKGN, and ONSET) shows a relatively consistent pattern. In each scenario, dewpoint and relative humidity variables show the highest degree of significance. Followed by variables for precipitation, showing a moderate degree of significance, and variables for temperature, soil–water and wind-gust showing the lowest degree of significance. Additionally, wind-gust is most significant at STGN, and precipitation is most significant at PKGN, and fewer variables are highly significant, and none are moderately significant at ONSET (Tables 2 and 3). This sequence of meteorological events agrees with the prior published research (Robinson et al., 2018) but also illustrates the conditions needed for a smog event to occur (Hernandez et al., 2017; Zhang et al., 2017).

**Fig. 4:**
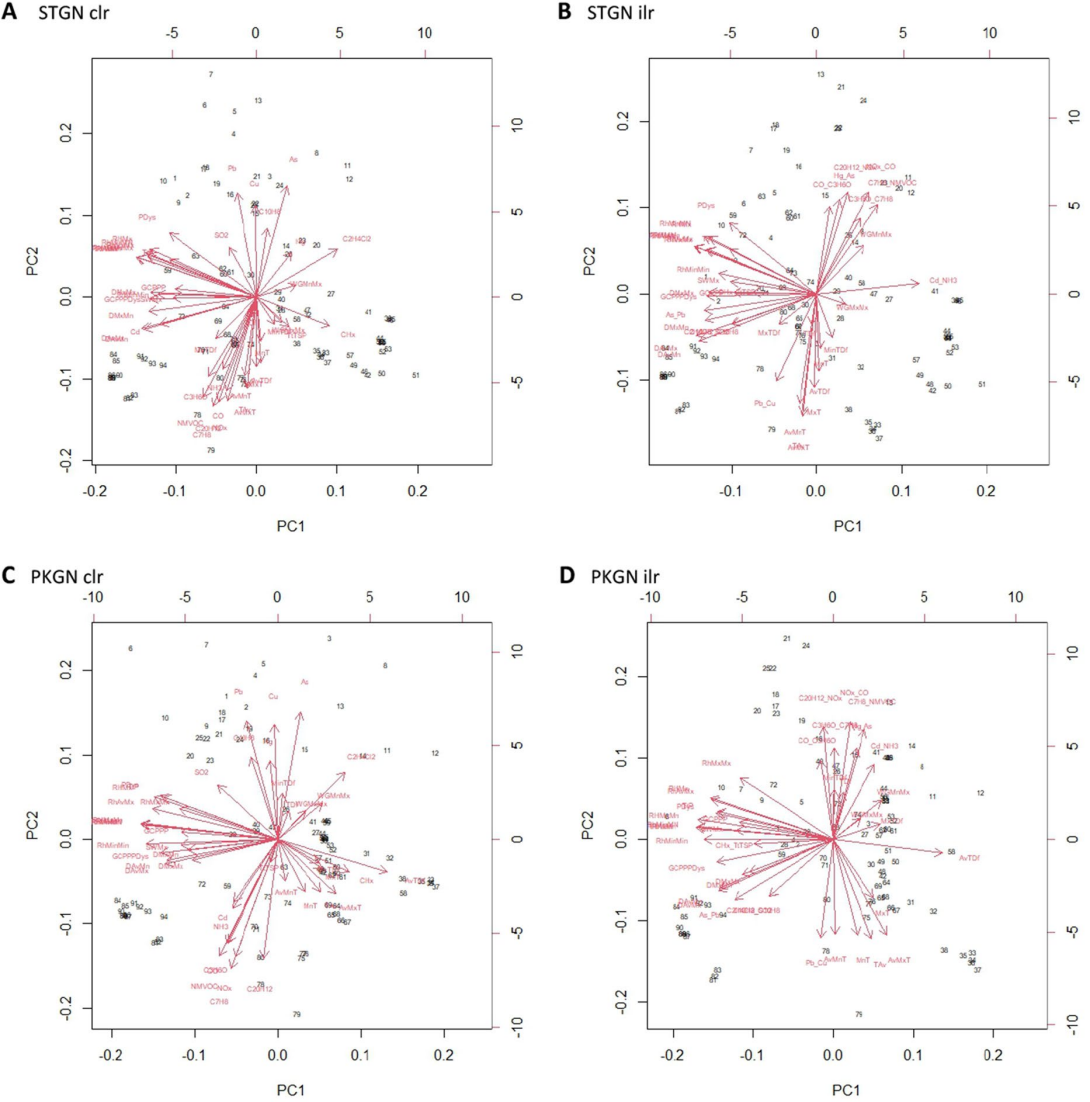
Principal Component Analysis (PCA) biplots for STGN (start of calving season), PKGN (peak of calving season), containing either clr or ilr transformed environmental pollutant variables and meteorological data from each event. Each plot contains principal component (PC) one and two and show associations between variables. The variables for humidity (RHUMID) and dewpoint (DEWP) are key for the interpretation of each plot, in **A** they cluster in the left and top left of the plot, in **B** the left and top left of the plot, in **C** the left of the plot, in **D** the left of the plot. Name key in supplementary material

**Fig. 5:**
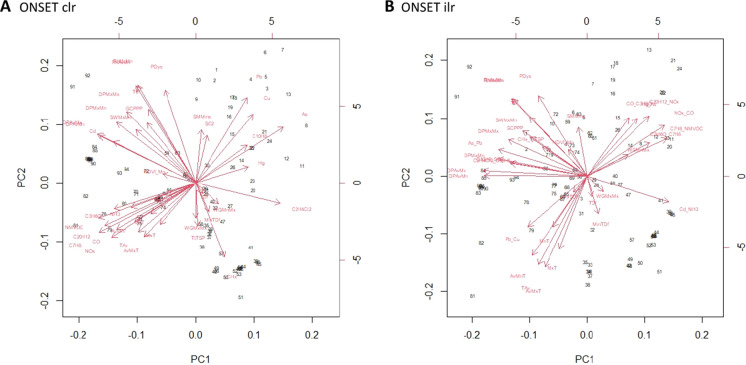
ONSET (onset of mass die-off) in containing either clr or ilr transformed environmental pollutant variables and meteorological data from each event. Each plot contains principal component (PC) one and two and show associations between variables. The variables for humidity (RHUMID) and dewpoint (DEWP) are key for the interpretation of each plot, **A** the bottom left and central part of the plot, and in **B** the bottom left and central part of the plot. Name key in supplementary material

The outputs from the PCAs show a similar pattern where meteorological variables can be assigned to each phase. Inferences about the synergistic combinations of environmental pollutants can also be considered. In the analysis, six PCAs were conducted, each was the clr and ilr transformed environmental pollutant data, separately analysed with the meteorological variables (Figs. 4 and 5). Principal components (PCs) 1–3 of each PCA were taken, and glm with a binomial distribution (logistic regression) was used to analyse the PCs, the same as the individual variables above (Table 4). These results should be interpreted carefully, one of the PCAs, ONSET ilr demonstrated no significant relationships with the mass die-off events. Each scenario: STGN, PKGN, and ONSET, will be considered and significant PCs discussed. The variable names are abbreviations of those above and a key is in the supplementary material. STGN clr PC2 showed moderate significance, with an association between temperature variables and pollutant variables: CO, NOx, C20H12, C7H8, NMVOC, C3H6O, and NH3, with wind gust associated with TtTSP. Furthermore, the pollutants SO2, Pb, Cu, C10H8, As, and Hg are also associated. For STGN ilr, PC1 showed no significance but was marginal (low AIC value), variables for dewpoint, precipitation, maximum soil water, minimum humidity, and maximum wind gust, were associated with showing a signal for a rain event. Also, the balances for As_Pb, CHx_TtTSP, Cd_NH_3_, C2H4Cl2 _ C10H8, and C10H8_SO_2_ were highlighted. This is a group of pollutants associated with both atmospheric particulates and soil pollutants. PC2 showed low significance, there is an association between temperature and mean-maximum wind gust and the balances for pollutants are also represented. Pb_Cu correlated with temperature. Whilst Hg_As, CO_C3H6O, C20H12_NOx, NOx_CO, C7H8_NMVOC, and C3H6O_C7H8, correlate with wind gust. These pollutants co-correlate and are associated with particulates, and soil pollution (Fig. 4).

**Table 4:** Logistic regression, *P*, and AIC values for PC 1–3 for each analysis of pollutant data and meteorological variables at the predicted start (STGN) and peak (PKGN) of the calving season, and onset (ONSET) of mass die-off

PCA Output	*P* value	AIC
PC1_STGN_clr_Pollutant	0.951	NA
PC2_STGN_clr_Pollutant	0.0027	59.597
PC3_STGN_clr_Pollutant	0.075	79.848
PC1_STGN_ilr_Pollutant	0.0715	10.434
PC2_STGN_ilr_Pollutant	0.00144	65.773
PC3_STGN_ilr_Pollutant	0.0901	80.227
PC1_PKGN_clr_Pollutant	0.00565	17.033
PC2_PKGN_clr_Pollutant	0.00179	64.51
PC3_PKGN_clr_Pollutant	0.0237	76.982
PC1_PKGN_ilr_Pollutant	0.00166	20.766
PC2_PKGN_ilr_Pollutant	0.000256	57.606
PC3_PKGN_ilr_Pollutant	0.695	82.969
PC1_ONSET_clr_Pollutant	0.00291	16.887
PC2_ONSET_clr_Pollutant	0.0671	79.652
PC3_ONSET_clr_Pollutant	0.132	80.847
PC1_ONSET_ilr_Pollutant	0.996	NA
PC2_ONSET_ilr_Pollutant	0.751	83.02
PC3_ONSET_ilr_Pollutant	0.207	81.549

*P* value < 0.05 indicates significance, AIC < 50 highly significant, AIC > 50 moderately significant, low degree of significance (significant *P* value and AIC value greater than 60), *P* value > 0.05 indicates insignificance

The PKGN scenario showed the highest level of overall significance compared with the other two scenarios. PKGN clr PCs 1–3 all showed significance, which is unusual. PC1 was highly significant and contained variables for meteorological variables: precipitation, dewpoint, soil–water, humidity, average temperature, and the pollutant CHx marginally. Whilst PC2 showed low significance and highlighted temperature, and a broad range of pollutants: Cu, Hg, Pb, As, and C10H6, correlate with minimum temperature difference. Whilst, Cd, TtTSP, NH3, CO, C3H6O, NMVOC, NOx, C7H8, and C20H12 correlating with average temperature variables. PC3 showed low significance and highlighted average temperature correlating with CHx, and SO_2_ remaining alone. In this PCA, PC1 highlights the rise in H_2_O present in the local environment (Kock et al., 2018). PC2 demonstrates a temperature change and the potential increase in the bioavailability of the pollutants listed. PC3 shows further temperature change and potential pollutant release. The PKGN ilr PCA shows a high degree of significance for PC1: precipitation, dewpoint, temperature difference, humidity, and CHx_TtTSP. There is a moderate degree significance for PC2: temperature variables correlating with Pb_Cu, and minimum temperature difference, correlating with Cd_NH_3_, Hg_As, CO_C3H6O, C3H6O_C7H8, C20H12_NOx, C7H8_NMVOC, and NOx_CO. The combination of variables in both PKGN PC1s. Shows the factors associated with a sudden rise in H_2_O and temperature required for a smog event (Hernandez et al., 2017; Zhang et al., 2017). PC2 shows the release of a variety of environmental pollutants into the local environment. Both combined would reflect the conditions needed for a toxic smog event (Fig. 4). Lastly, ONSET clr showed a higher degree of significance for PC1, and highlights: mean NDVI, Hg, and Dichloroethane /C2H4Cl2 primarily, as well as variables for dew point average and Cd, with temperature variables correlating with C3H6O, NH3, NMVOC, C20H12, C7H8, CO, and NOx. Overall, suggesting a toxic smog event, formed by humidity and small particulates, and the resultant release of a variety of environmental pollutants (Hernandez et al., 2017; Zhang et al., 2017). ONSET ilr showed no significance (Fig. 5, Table 4). The central inference from this analysis, during the mass die-off event, is that there was not a strong association with wind-gust, but a strong indication of a precipitation event. Followed by a rise in temperature, and the resultant high humidity. The shift in significance from PCs of both clr and ilr in STGN and PKGN, too PCs from clr transformed data at ONSET. Hints at an environmental transition. From a complex mixture to dissociated components, but this is speculative. Additionally, though unbalanced, there are strong associations between temperature, resultant humidity, and various environmental pollutants. Which would be released into the environment from an imprinted signal. Created by the aeolian deposition of environmental pollutants. Therefore, the build-up of pollutants in soils (source), and the precipitation event. Then followed by humidity, and the potential release of environmental pollutants into a smog (pathway), which is largely in agreement with the environmental mechanism proposed in Fig. 1. Suggesting the sequence of events agrees with those for mass die-off mechanism proposed in Fig. 2 (Fey et al., 2015; Kock et al., 2018).

### Environmental pollutants, smog, and saiga antelope physiology

It should be reiterated that Saiga antelope have a highly specialised nasal anatomy. Adaptations for dealing with climatic extremes, warming air in winter, cooling in summer, and filtering particulates from the desert environment (Clifford & Witmer, 2004; Frey et al., 2007)^.^ Previously, work on Saiga antelope has not indicated that the bioaccumulation of pollutants in the environment has impacted their populations (Kock et al., 2018). However, mass die-off events are not chronic outcomes. Characteristically caused by the steady accumulation of an agent over an extended period of time, but acute events. Caused by a synergy of multiple factors coalescing to force a change in the environment. Often sudden and irreversible, having deadly outcomes for the populations caught in its path (Fey et al., 2015). Our findings highlight the ecotoxicological implications of a perfect storm. A smog formed from environmental toxins and adverse weather, that likely coalesced to cause mass die-off in Saiga antelope.

### Mass die-offs in space and time

Mass die-off events are caused by different factors. Other climate change-induced examples include temperature fluctuations in lakes and seas (Romano et al., 2020; Till et al., 2019) hypoxia in coral atolls (GajdzikLaura, 2017) and the spread of novel diseases in dense populations (Bacharach et al., 2016; Miaud et al., 2016). Synergisms between environmental factors and disease have also occurred. In the Serengeti, the African lion (*Panthera leo*) population was reduced by a third in 1994, from a combination of drought and disease caused by Babesia from cape buffalo (*Syncerus caffer*) and latent canine distemper (*Canine morbillivirus*) (Munson et al., 2008). In the Western Mediterranean between 1981 and 2004, mass die-off events occurred in the striped dolphin (*Stenella coeruleoalba*) from a combination of bioaccumulated polychlorinated biphenyls, and infections of the phoront cirriped *Xenobalanus globicipitis*, and the mesoparasitic copepod *Pennella balaenopterae* (Aznar et al., 2005). In the fossil-record mass die-off events can be observed and attributed to natural environmental phenomena. Like drought (> 1000 *Coelophysis*, ghost ranch New Mexico) (Schwartz & Gillette, 1994), drowning (*Centrosaurus*, bone bed 43 Alberta, Canada) (Ryan et al., 2001), flash-flooding (*Hypsilophodon*, Isle of Wight, UK) (Coram et al., 2017) and miring (*Plateosaurus, sites in* Germany and Switzerland) (Sander, 1992). The modern-day (Anthropocene) examples show events linked to both climate change and synergistic activity between environmental pollution and disease. Whist the Mesozoic examples document natural events, denoting a recent shift to novel anthropogenic phenomena as the primary cause of mass die-off phenomena. In future, anthropogenic phenomena will play an increased role in how mass die-offs occur and perpetuate. 

#### Industrial and environmental phenomena

The environmental pollutants in this study can be linked to their most likely sources. Pb, Hg, Cu, and As, are likely from mining or industrial sources. Naphthalene (C10H8), Dichloroethane (C2H4Cl2), and SO_2_ are likely from the combustion of fossil fuels and automobiles. Cd and NH_3_ from agricultural sources. Toluene (C7H8), Benzo(α)pyrene (C20H12), Acetone (C3H6O), NOx, and CO could be from paint thinner, industrial chemical processes, or incomplete combustion (Kerimray et al., 2020). Kazakhstan is a rapidly industrialising nation, and research on pollutants, smog, soil accumulation, and public health concerns has increased (Iztileu et al., 2013). In the future, as in much of the Anthropocene, measures will need to be taken to manage and mitigate against the impacts of environmental pollution, and climate change will only extricate the circumstances that lead to the events described within Fig. 1.

## Conclusion

In this scenario, pollutants accumulated in desert soils (source), and were released quickly by a sudden precipitous weather event. These pollutants were activated in the environment by elevated temperatures, forming a volatile smog (pathway), and were respired by the saiga antelope (receptor) (Fig. 1). Under normal circumstances when the herds are migrating, it is possible the herds would simply move-on or resist the smog’s effects. However, in the calving season, the adults, especially females, are likely to be immuno compromised. So, the herd arrives at the calving ground, a sudden smog occurs, having an especially acute impact on the specialised noses of the Saiga. Thus, weakening the animals yet further. The *P*. *multocida* serotype B infection begins to spread, and the animals die *en masse*. Singularly, each of these variables, are factors the Saiga could withstand. Combined within this pollutant linkage model, the animals are pushed into an acute state of ill health, causing a mass die-off event to occur (Fig. 2). It should be noted that this analysis is drawing on disparate sources and is reliant on a compositional aeolian deposition signal. experimental work, field observation, and surveying would be required to clarify these concepts such as the deployment of stationary sensors to measure pollutant levels at these sights when high humidity occurs. Further experimental work on the potentially troubling nasal anatomy of the Saiga Antelope may also demonstrate the effects of gaseous pollutants on these animals.

### Supplementary Information

Below is the link to the electronic supplementary material.

Supplementary file1 (ODT 231 KB)

## Data Availability

PCA key and outputs can be seen in the supplementary material. Data for Saiga Antelope and meteorological variables can be found at: 10.5285/912ea336-ac90-418f-be6a-7ae226e167e9, and air pollution can be found at: https://www.gov.kz/memleket/entities/economy?lang=en. Derived data, model coefficients, odds ratios, confidence intervals, and code for this analysis can be seen in 10.17632/2sph9gygwp.1.
