# Supplementary material for: A nose for trouble: ecotoxicological implications for climate change and disease in Saiga antelope (S. t. tatarica)
Source: Environ Geochem Health. 2024 Feb 17;46(3):93. doi: 10.1007/s10653-024-01874-y (PMC10874336; doi:10.1007/s10653-024-01874-y)
Supplement: Supplementary file 1 [file 10653_2024_1874_MOESM1_ESM.odt]

Supplementary Material

PCA outputs for each set of PCA analyses and keys for all variables. 

Table 1: STGN Key 

| Logistic STGN | STGN PCA |
| --- | --- |
| TOTPRECIP_10D_STGN | TtP |
| GCPP_PRECIP_10D_STGN | GCPPP |
| PRECIPDAYS_10D_STGN | PDys |
| GCPP_PRECIPDAYS_10D_STGN | GCPPPDys |
| SOILWATER1_MAXMN10D_STGN | SWMx |
| MINTEMP_10D_STGN | MnT |
| MAXTEMP_10D_STGN | MxT |
| TEMPAV_10D_STGN | TAv |
| AVOFMINTEMP_10D_STGN | AvMnT |
| AVOFMAXTEMP_10D_STGN | AvMxT |
| AVTEMPDIFF_DAY_10D_STGN | AvTDf |
| MXTEMPDIFF_DAY_10D_STGN | MxTDf |
| TEMPDIFF_MINS_10D_STGN | MinTDf |
| DEWPAVMN_10D_STGN | DAvMn |
| DEWPMXMN_10D_STGN | DMxMn |
| DEWPAVMX_10D_STGN | DAvMx |
| DEWPMXMX_10D_STGN | DMxMx |
| RHUMID_MEAN10D_STGN | RHMn |
| RHUMID_MAXOFMN10D_STGN | RHMx |
| AVRHUMID_MAX10D_STGN | RhAvMx |
| AVRHUMID_MEAN10D_STGN | RHAvMn |
| MAXRHUMID_MEAN10D_STGN | RHMxMn |
| MAXRHUMID_MAX10D_STGN | RhMxMx |
| MINRHUMID_MEAN10D_STGN | RhMinMN |
| MINRHUMID_MIN10D_STGN | RhMinMin |
| WINDGUST_MNOFMAX10D_STGN | WGMnMx |
| WINDGUST_MXOFMAX10D_STGN | WGMxMx |
| DIFFTEMP_10DAY_STGN | TDf |

Table 2: PKGN Key. 

| Logistic PKGN | PKGN PCA |
| --- | --- |
| TOTPRECIP_10D_PKGN | TtP |
| GCPP_PRECIP_10D_PKGN | GCPPP |
| PRECIPDAYS_10D_PKGN | PDys |
| GCPP_PRECIPDAYS_10D_PKGN | GCPPPDys |
| SOILWATER1_MAXMN10D_PKGN | SWMx |
| MINTEMP_10D_PKGN | MnT |
| MAXTEMP_10D_PKGN | MxT |
| TEMPAV_10D_PKGN | TAv |
| AVOFMINTEMP_10D_PKGN | AvMnT |
| AVOFMAXTEMP_10D_PKGN | AvMxT |
| AVTEMPDIFF_DAY_10D_PKGN | AvTDf |
| MXTEMPDIFF_DAY_10D_PKGN | MxTDf |
| TEMPDIFF_MINS_10D_PKGN | MinTDf |
| DEWPAVMN_10D_PKGN | DAvMn |
| DEWPMXMN_10D_PKGN | DMxMn |
| DEWPAVMX_10D_PKGN | DAvMx |
| DEWPMXMX_10D_PKGN | DMxMx |
| RHUMID_MEAN10D_PKGN | RHMn |
| RHUMID_MAXOFMN10D_PKGN | RHMx |
| AVRHUMID_MAX10D_PKGN | RhAvMx |
| AVRHUMID_MEAN10D_PKGN | RHAvMn |
| MAXRHUMID_MEAN10D_PKGN | RHMxMn |
| MAXRHUMID_MAX10D_PKGN | RhMxMx |
| MINRHUMID_MEAN10D_PKGN | RhMinMN |
| MINRHUMID_MIN10D_PKGN | RhMinMin |
| WINDGUST_MNOFMAX10D_PKGN | WGMnMx |
| WINDGUST_MXOFMAX10D_PKGN | WGMxMx |
| DIFFTEMP_10DAY_PKGN | TDf |

Table 3: ONSET Key. 

| Logistic ONSET | ONSET PCA |
| --- | --- |
| MAXRHUMID_MEAN10D_STGNONSET | RhMxMn |
| AVRHUMID_MEAN10D_STGNONSET | RhAvMn |
| DEWPAVMX_10D_STGNONSET | DPAvMx |
| DEWPMXMN_10D_STGNONSET | DPMxMn |
| DEWPMXMX_10D_STGNONSET | DPMxMx |
| DEWPAVMN_10D_STGNONSET | DPAvMn |
| SOILWATER1_MAXMN10D_STGNONSET | SWMxMn |
| DIFFTEMP_10DAY_STGNONSET | TDf |
| WINDGUST_MNOFMAX10D_STGNONSET | WGMnMx |
| WINDGUST_MXOFMAX10D_STGNONSET | WGMxMx |
| GCPP_PRECIP_10D_STGNONSET | GCPPP |
| TOTPRECIP_10D_STGNONSET | TtP |
| PRECIPDAYS_10D_STGNONSET | PDys |
| MAXTEMP_10D_STGNONSET | MxT |
| AVOFMAXTEMP_10D_STGNONSET | AvMxT |
| TEMPAV_10D_STGNONSET | TAv |
| MINTEMP_10D_STGNONSET | MnT |
| AVOFMINTEMP_10D_STGNONSET | AvMnT |
| MXTEMPDIFF_DAY_10D_STGNONSET | MxTDf |
| SNOWMELT_DAYONSET_STGNONSET | SMMins |
| TEMPDIFF_MINS_10D_STGNONSET | MinTDf |
| NDVI_MEAN15D_STGNONSET | NDVI_Mn |

Table 4: Pollutant Key. 

| Logistic Pollutant | Pollutant PCA |
| --- | --- |
| Pb_clr | Pb |
| Cd_clr | Cd |
| Hg_clr | Hg |
| Cu_clr | Cu |
| As_clr | As |
| Toluene_clr | C7H8 |
| Benzo_a_pyrene_clr | C20H12 |
| Naphthalene_clr | C10H8 |
| Dichlorethane_clr | C2H4Cl2 |
| Acetone_clr | C3H6O |
| SO2_clr | SO2 |
| NOx_clr | NOx |
| NMVOC_clr | NMVOC |
| NH3_clr | NH3 |
| CO_clr | CO |
| CHx_clr | CHx |
| TSP_total_clr | TtTSP |
| Hg_As_ilr | Hg_As |
| As_Pb_ilr | As_Pb |
| Pb_Cu_ilr | Pb_Cu |
| Dichlorethane_Naphthalene_ilr | C2H4Cl2_C10H8 |
| Naphthalene_SO2_ilr | C10H8_SO2 |
| Cd_NH3_ilr | Cd_NH3 |
| CHx_TSP_ilr | CHx_TtTSP |
| Benzo_a_pyrene_NOx_ilr | C20H12_NOx |
| NOx_CO_ilr | NOx_CO |
| CO_Acetone_ilr | CO_C3H6O |
| Acetone_Toluene_ilr | C3H6O_C7H8 |
| Toluene_NMVOC_ilr | C7H8_NMVOC |

Chart, scatter chart

Description automatically generated

Fig. 1: Biplot STGN clr Pollutant.

Chart, scatter chart

Description automatically generated

Fig. 2: PCA STGN clr Pollutant.

Chart, histogram

Description automatically generated

Fig. 3: Scree STGN clr Pollutant.

Chart, scatter chart

Description automatically generated

Fig. 4: Biplot STGN ilr Pollutant.

Chart, scatter chart

Description automatically generated

Fig. 5: PCA STGN ilr Pollutant.

Chart, histogram

Description automatically generated

Fig. 6: Scree STGN ilr Pollutant.

Chart, scatter chart

Description automatically generated

Fig. 7: Biplot PKGN clr Pollutant.

Chart, scatter chart

Description automatically generated

Fig. 8: PCA PKGN clr Pollutant.

Chart, histogram

Description automatically generated

Fig. 9: Scree PKGN clr Pollutant.

Chart, scatter chart

Description automatically generated

Fig. 10: Biplot PKGN ilr Pollutant.

Chart, scatter chart

Description automatically generated

Fig. 11: PCA PKGN ilr Pollutant.

Chart, histogram

Description automatically generated

Fig. 12: Scree PKGN ilr Pollutant.

Chart, scatter chart

Description automatically generated

Fig. 13: Biplot ONSET STGN clr Pollutant.

Chart, scatter chart

Description automatically generated

Fig. 14: PCA ONSET STGN clr Pollutant.

Chart, histogram

Description automatically generated

Fig. 15: Scree ONSET STGN clr Pollutant.

Chart, scatter chart

Description automatically generated

Fig. 16: Biplot OSNET STGN ilr Pollutant.

Chart, scatter chart

Description automatically generated

Fig. 17: PCA ONSET STGN ilr Pollutant.

Chart, histogram

Description automatically generated

Fig. 18: Scree ONSET STGN ilr Pollutant.
